# Diagnostic Accuracy of Screening Tests for Diabetic Peripheral Neuropathy Among Outpatient Attendees of Follow-Up Care for Diabetes in Central India

**DOI:** 10.7759/cureus.91706

**Published:** 2025-09-06

**Authors:** Kalaiselvi Selvaraj, Pradeep Deshmukh, Mrunal Phatak, Ashlesh Patil, Lena Charlette, Rajashree Khot, Mubashshera F Khan, Srinivasan Thanigachalam

**Affiliations:** 1 Community and Family Medicine, All India Institute of Medical Sciences, Madurai, Madurai, IND; 2 Community Medicine, All India Institute of Medical Sciences, Nagpur, Nagpur, IND; 3 Physiology, All India Institute of Medical Sciences, Nagpur, Nagpur, IND; 4 General Medicine, All India Institute of Medical Sciences, Nagpur, Nagpur, IND

**Keywords:** biothesiometry, diabetic foot, diabetic neuropathy, diagnostic accuracy, monofilament

## Abstract

Background and objectives

Of the several screening tests used in the screening of diabetic peripheral neuropathy (DPN), each has its demerits, which pose a threat to reliability. There is no single optimal method for the early diagnosis of DPN. This study was conducted to compare the diagnostic accuracy of monofilament, biothesiometer, and symptom-based scoring in detecting DPN, to determine the optimal cut-off for biothesiometer score compared to nerve conduction studies (NCS), and to compare the time taken to screen with monofilament and biothesiometer.

Material and methods

This cross-sectional, comparative, diagnostic accuracy study was conducted in a tertiary care institute in Central India. Among the patients with diabetes mellitus (DM), those who were advised NCS between March and October 2023 were included in the study.

Results and discussion

A total of 78 limbs among 39 patients with diabetes were assessed for DPN. The sensitivity of clinical scoring, monofilament, and biothesiometer (cut-off = 10) was 15.6% (5.3-32.8), 35.5% (23.7-48.7), and 71% (58.1-81.8), respectively. A biothesiometer score of 10 was identified as optimal, with an area under the curve of 0.7132 (0.56-0.87), and the time taken to administer the biothesiometer was about 2.5 times more compared to the monofilament assessment.

Conclusions

Biothesiometry has higher sensitivity compared to other screening methods. The biothesiometer cut-off at 10 had higher sensitivity compared to the existing standards of 15mV. The need for a lower cut-off for biothesiometry has to be established with reference to NCS standards in larger studies.

## Introduction

Chronic sensorimotor distal symmetric polyneuropathy and autonomic neuropathy are the most common complications of diabetes mellitus (DM) [1]. Diabetic peripheral neuropathy (DPN) most commonly affects small and large fibers. It may be present in 10-15% of people living with diabetes at the time of diagnosis, and the prevalence increases to up to 50% within 10 years of diagnosis [2,3]. DPN is a risk factor for developing diabetic foot, which includes infections, ulceration, gangrene, and Charcot neuropathic osteoarthritis [4]. The prevalence of diabetic foot ulcers (DFUs) globally was 6.4% [5], and the lifetime incidence of developing DFUs was 15% among people living with diabetes [6]. Lower limb amputations were performed in 18% of DFU patients, and the survival was reduced to 50% at five years [7,8].

In about half of the patients with DPN, the condition is asymptomatic, and hence screening plays a crucial role in early diagnosis and prevention of progression of the disease. The American Diabetes Association recommends screening for DPN annually, starting at the time of diagnosis of type 2 diabetes and after five years of type 1 diabetes. The tests assess the temperature or pinprick sensation (small-fiber function), vibration sense using a 128-hertz tuning fork (large fiber function), and 10g monofilament testing to identify the risk of diabetic ulcers and amputation (loss of protective sensation) [9].

Nerve conduction studies (NCS) are the gold standard in diagnosing large fiber neuropathy, but are not readily available, expensive, time-consuming, and require trained examiners [10-12]. Several screening tools are available for the early diagnosis of diabetic neuropathy. The scored clinical tools, such as the Michigan Neuropathy Screening Instrument (MNSI), Toronto Clinical Neuropathy Score (TCNS), and United Kingdom Screening Test (UKST), are found to be valid for screening; however, the cutoff points are varied [12-14]. Likewise, the diagnostic accuracy of the 10g monofilament test is highly variable [12,15] and is affected by the monofilament used, the method of performing the test (sites), thresholds, and subjective differences [12,16]. Of the various types of nerve functions, vibration sense is lost the earliest [17]. Vibration perception thresholds (VPTs) are tested using various methods such as a tuning fork, biothesiometer, and vibratip. Quantitative VPT testing provides objective results, but the thresholds vary according to patient age, sex, and height [12,18].

Thus, there is no single optimal method for the early diagnosis of DPN [12]. The sensitivity and specificity of the different tests at different thresholds have not been studied in India. There is little evidence that indicates the need for different cut-offs among the Indian population based on age, ethnicity, and the thickness of the sole [19,20]. Hence, we felt that establishing the Indian-specific cutoff could be a need. So, this study was conducted to compare the diagnostic accuracy of monofilament, biothesiometer, and symptom-based scoring in detecting DPN, to determine the optimal cut-off for biothesiometer score compared to NCS, and to compare the time taken to screen with monofilament and biothesiometer.

This article was previously posted to the Research Square preprint server on December 18, 2024.

## Materials and methods

Study design and setting

This cross-sectional, comparative, diagnostic accuracy study was conducted in a tertiary care institute in Central India. In the institute, all adults (>18 years) with diabetes are treated in the department of internal medicine, and routine screening tests for DPN are not conducted in asymptomatic patients. In symptomatic patients, the Michigan patient symptom checklist is administered, followed by inspection for foot deformities, lesions, vibration tests using a biothesiometer, and monofilament tests. In parallel, NCS is conducted in the human physiology lab.

Sample size

This study was nested within a larger cohort of 2007 patients, aimed to assess the cost-effectiveness of the biothesiometer and monofilament.

The sample size estimation was done using the Statistics and Sample Size mobile application (Truc Thanh Thai, Ho Chi Minh City, Vietnam) for the parent study. The formula used was based on estimating sensitivity, where *d* is the absolute precision, *Z* is the value for 95% CI, and *Sens* is the sensitivity of the biothesiometer. The formula is as follows:

N = [Z(1-α/2)]² × Sens × (1 - Sens) / [d² × Prevalence]

The sample size was calculated to be 384 (~400) patients with DPN, based on the positivity rate by biothesiometer as 48.4% and 17.5% by monofilament test [21], 5% absolute precision, and 5% alpha error. ​Considering the prevalence of DPN as 20%, a total of 1920 (~2000) diabetic individuals were planned for enrolment. The estimation of diagnostic accuracy measures against the gold standard NCS was added for a limited subset of patients. Among the patients with DM enrolled in the parent study, those who were advised NCS were included in the study.

Study population and sampling

Patients living with diabetes, aged 18 years and above, visiting the outpatient department (OPD) of the department of internal medicine or general surgery with or without any complications were included, irrespective of the duration of diagnosis and symptoms. However, patients without any data related to any screening or NCS were excluded. The reference period for patient enrolment was between March and October 2023. The duration of disease among enrolled patients ranged from two months to 24 years. Patients with type 1 diabetes and amputation in any part of the leg, which precludes the administration of screening tests for DPN, were excluded. As the standard of care and follow-up care for complications of screening would be different for type 1 and type 2 diabetes, and more than 97% of cases that are catered in these settings are of type 2 DM, we preferred to exclude type 1 DM patients. In the internal medicine OPD, a trained medical technician administered a structured proforma including the Michigan neuropathy symptom checklist and examination using a biothesiometer. Following this, the patient was sent to the nurses' station, where the monofilament test was conducted. For a subset of 39 patients, NCS was advised by the physician, and their results were compared with the above tests in this study.

Training for Accessor and Standardization

A medical technician who had undergone a three-year bachelor's degree in laboratory science was enrolled in the study for the assessment. Similarly, the nurse who was available at the OPD of the general medicine registration counter was involved in the study. The technician received training from the principal investigator. After the demonstration, the technician was allowed to perform independently on volunteers. When the investigators felt all the steps were followed appropriately, she was allowed to perform independently on patients with diabetes. The investigators involved in the study observed up to 20 patients and conducted random on-site assessments of up to five patients/month. The dorsum of the hand was used to make the patient understand the difference between touch and vibration.

This study was carried out in the outpatient setting. In this setup, all patients are initially checked by the nurse for all vital parameters. In this counter, the monofilament test was carried out. In the adjacent room, the technician performed the biothesiometer test just before their consultation with the physician. The time duration between monofilament and biothesiometry would not exceed more than 15 minutes.

Screening for DPN by the Michigan neuropathy patient symptom score

In the present study, we obtained formal permission from the original copyright holders to use the MNSI and administered the validated 15-item patient version of the scale [22]. Symptom for each leg was captured separately. Affirmative responses in most items were scored as one, except for items 7 ( able to feel hot and cold water) and 13 (able to sense the leg while walking). These two items were scored in reverse. Cumulatively, patients with a score of seven or more were classified as high risk for DPN [13].

Screening for DPN by the 10g monofilament test

The monofilament test was done using Semmes-Weinstein 10g monofilament, which generates 10g force when it buckles (Monofilament 10g/5.07, Kodys Diabetic Foot Care Products, Kody Medical Electronics Pvt Ltd., Chennai, India). After sensitizing the patients to the touch sensation by monofilament over their hands, with eyes closed, the actual assessment was done at six sites on the plantar aspect of the feet, namely, the ball of the great toes, the metatarsal head of the first, third, and fifth toes, the middle of the medial border of the foot, and the heel. A detailed description of screening by the monofilament test is given in the Appendices. A score of 1 was assigned for each area if the patient could feel the sensation of the monofilament. The cumulative score was derived for each leg separately. If the total score is ≤4 in a foot, the foot is considered "at risk." In other words, if the patient is not able to feel the sensation in two or more points in one leg, it is classified as DPN.

Screening for DPN by biothesiometer

Patients were sensitized to understand the vibratory sensation by placing the biothesiometer (Kodys, Digital Biothesiometer Model Biothezi VPT, Kody Medical Electronics Pvt Ltd.) probe over the bony prominence in their hands and to differentiate the vibratory feel from the pressure sensation. After the sensitization, with eyes closed, the assessment was done at six places over the plantar aspects of the feet, namely, the ball of the great toes, the metatarsal head of the first, third, and fifth toes, the middle of the medial border of the foot, and the heel.

The vibratory threshold was initially set with the vibratory sensation threshold perceived over the hands. It was then gradually increased till the patient expressed that they could feel the vibratory sensation. The threshold level was reconfirmed by reducing the vibration levels and assessing patient perception. In each area, the vibratory threshold shown on the screen of the biothesiometer was noted.

The average score of the vibratory threshold was calculated for each leg (cumulative score/six or number of sites tested). In case any of the sites were not assessed, the average score was estimated based on the number of sites tested. Signs of neuropathy were classified based on the categories specified by the manufacturers. Based on the receiver operating characteristic (ROC) curve plotted between true positive and false positive at each score, an optimum cut-off was identified. Overall, an average score beyond the optimum cut-off identified from the ROC was considered positive for DPN. A detailed description of screening with the biothesiometer is given in the Appendices.

Assessment for DPN based on NCS

NCS was done using an electromyography/electrophysiology/NCS measuring system (MYOQUICK, Micromed, Treviso, Italy). Sensory and motor NCS were done in the bilateral median, ulnar, radial, peroneal, tibial, and sural nerves. Sensory NCS were done using the antidromic method. F-wave studies were done for the bilateral median, ulnar, and tibial nerves, and H-reflex studies for the bilateral tibial nerves. Supramaximal stimulation was used in all settings, except for the H-reflex, where the stimulation was gradually increased till the appearance of a good amplitude M wave. The parameters recorded include distal latencies (DL), amplitudes of compound motor action potentials (CMAP), duration of CMAP, F wave latencies, and conduction velocities (CV) in motor nerves. In sensory nerves, latencies and amplitudes of the sensory nerve action potentials and their conduction velocities were documented. For the H-reflex, the latencies and amplitude were noted. The patient-level preparatory steps followed for NCS are given in the Appendices.

*Classifying the State of Diabetic Peripheral Neuropathy Using *NCS

The NCS parameters were compared with laboratory norms, as well as opposite side comparison during interpretation. The axonal condition was labeled based on amplitude reduction below the lower limit of normal, with no or little effect on conduction velocities and distal latencies. To identify a chronic demyelinating condition, we assessed motor nerves for at least three specific criteria [23]: (1) prolonged DL in two or more nerves, excluding entrapment sites, where the DL is greater than 130% of the upper limit of normal; (2) CV slowing in two or more nerves, not between entrapment sites, with a CV less than 75% of the lower limit of normal; (3) for prolonged late responses, F waves and H reflexes in one or more nerves exceed 130% of the upper limit of normal. If the distal CMAP amplitude was very low, the absence of F waves may not be considered abnormal.

Additionally, we investigated for conduction block or temporal dispersion in one or more nerves. An unequivocal conduction block was identified by a proximal/distal CMAP area ratio of less than 0.50, while a possible conduction block was indicated by a proximal/distal CMAP amplitude ratio of less than 0.70.

Data analysis

Characteristics of patients are summarized as frequency, percentages (categorical variables), and mean and standard deviation (continuous variables). Diagnostic accuracy measures, namely, sensitivity, specificity, positive predictive value, and negative predictive value, are summarized as percentages with 95% confidence intervals. To identify the reliable cut-off and accuracy, the ROC curve was plotted for various biothesiometry values against the gold standard NCS. True positive and false positive proportions for each observed biothesiometry score were plotted. Based on Youden's index, the optimum cut-off, which gave a trade-off between sensitivity and specificity, was chosen. The time taken to complete the assessment for monofilament and biothesiometer is summarized as mean and standard deviation, and statistical significance for time comparison was done using a paired t-test. All analyses were performed using Stata software (StataCorp LLC, College Station, TX).

Ethical issues

Administrative approval (gatekeeper consent) was obtained from the hospital authorities of the tertiary care medical institute. Written Informed consent was obtained from all eligible patients enrolled in the study. The database was kept anonymized with access restricted only to the study team. Those who were found to have evidence of DPN were referred to their consulting physician for further management.

## Results

Characteristics of the patients

A total of 78 limbs among 39 patients with diabetes were assessed for DPN with clinical neuropathy scoring, monofilament, biothesiometer, and NCS. About 74% of the patients were diagnosed with diabetes after the onset of clinical symptoms or complications. More than 97% received treatment from the study site, which is a public tertiary care facility. Hypertension was the most common co-existent comorbidity, followed by cardiovascular diseases and stroke. Characteristics of the patients are given in Table 1.

**Table 1:** Characteristics of the study population (n = 39).

Characteristics	Frequency (%)
Age in years (mean ± SD)	56.9 ± 10.9
Duration of diabetes in years, median (IQR)	5 (2-10)
Circumstances in which diabetes mellitus was diagnosed	
During the work-up of symptoms	27 (69.2)
Manifested as complications	2 (5.1)
For surgery, pre-anesthetic assessment	1 (2.6)
Opportunistic screening	5 (12.8)
Not sure	4 (10.2)
Place of diabetes diagnosis	
Govt. primary/secondary facilities	4 (10.2)
Govt. tertiary care facilities	7 (19.9)
Private facilities	16 (40.1)
Others	18 (46.2)
Not sure	1 (2.6)
Place of treatment	
Govt. tertiary care facility	38 (97.4)
Private	1 (2.6)
Presence of co-morbidities	
Hypertension	27 (69.2)
Peripheral vascular disease	2 (5.1)
Cardiovascular disease	11 (28.2)
Cerebrovascular disease	7 (18)
Thyroid disorders	2 (5.1)
Chronic renal failure	1 (2.6)
Chronic musculoskeletal disorders	1 (2.6)
Chronic skin disorders	1 (2.6)
Impaired vision	4 (10.3)

The predominant symptoms of the patients with DPN were feeling hurt in the leg, pricking, and numbness (45-48%), followed by a burning sensation in the foot (33.3%) (Table 2).

**Table 2:** Distribution of pattern of symptoms reported in the Michigan neuropathy scale (N = 39).

Feature	Frequency (%)
Numbness in the leg	18 (46.2)
Burning sensation in the feet	13 (33.3)
Sensitive foot	7 (17.9)
Cramps	16 (41.0)
Pricking	19 (48.7)
Able to sense hot and cold sensations	39 (100)
Soreness in the leg	3 (7.7)
Provider diagnosed foot conditions	7 (17.9)
Weakness in the leg	22 (56.4)
Nocturnal symptoms in the leg	10 (25.6)
Hurt in the leg	19 (48.7)
Sense the foot while walking	37 (94.9)
Dryness	8 (20.5)
History of amputation	0 (0)
Examination (signs)	
Foot ulcer	1 (2.6)
Fissure & callus	6 (15.4)
Ankle reflux absent/reinforcement	3 (7.7)

**Figure 1:**
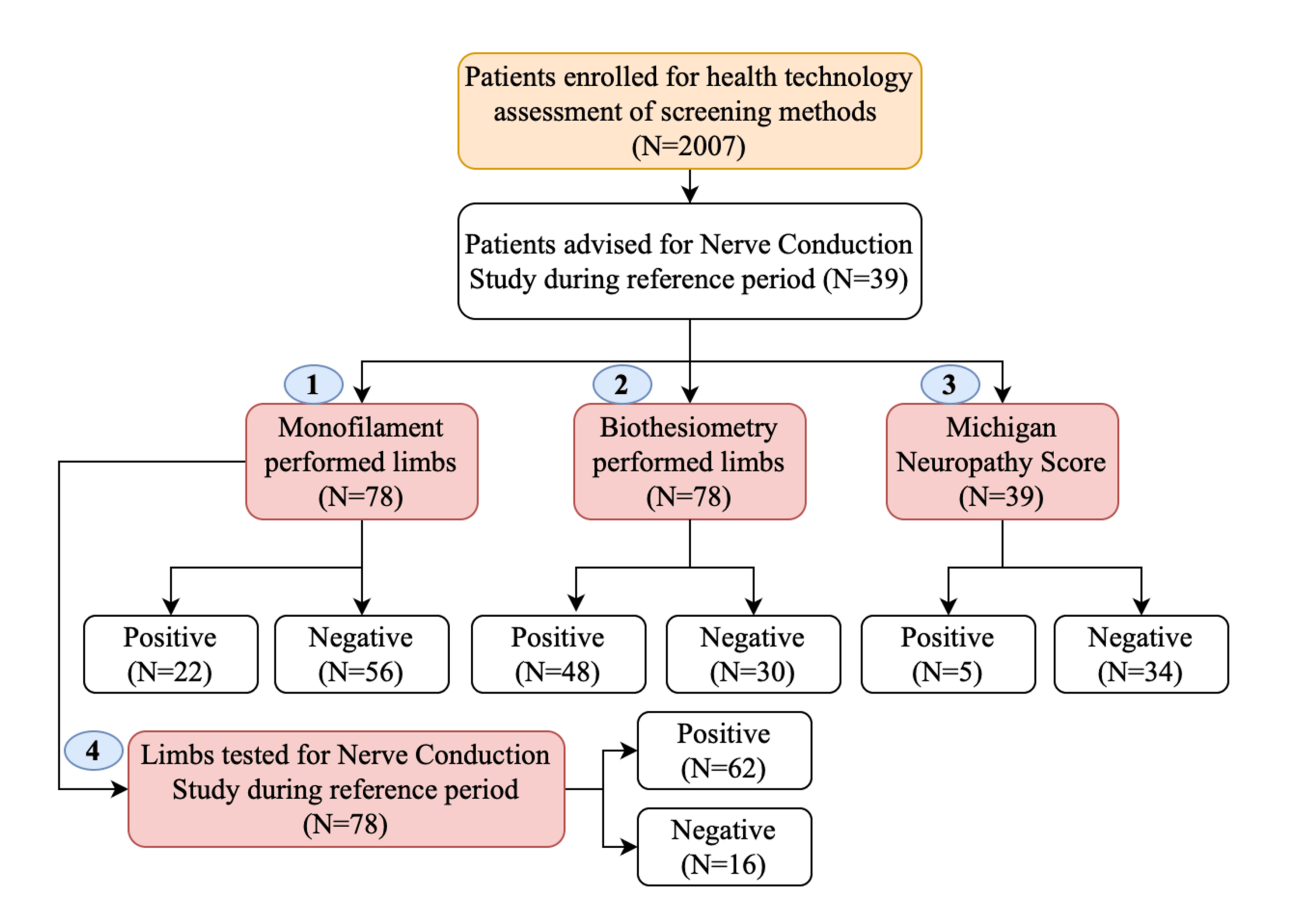
Patient flow and sequence of different tests performed among patients with diabetes enrolled for diagnostic accuracy of screening methods against nerve conduction study in Central India (2023).

The patient flow and sequence of screening methods performed in this study are depicted in Figure 1. No test was shown to be indeterminate, and there was no attrition or missing data among patients subjected to different tests.

The optimum cut-off for biothesiometry

**Figure 2:**
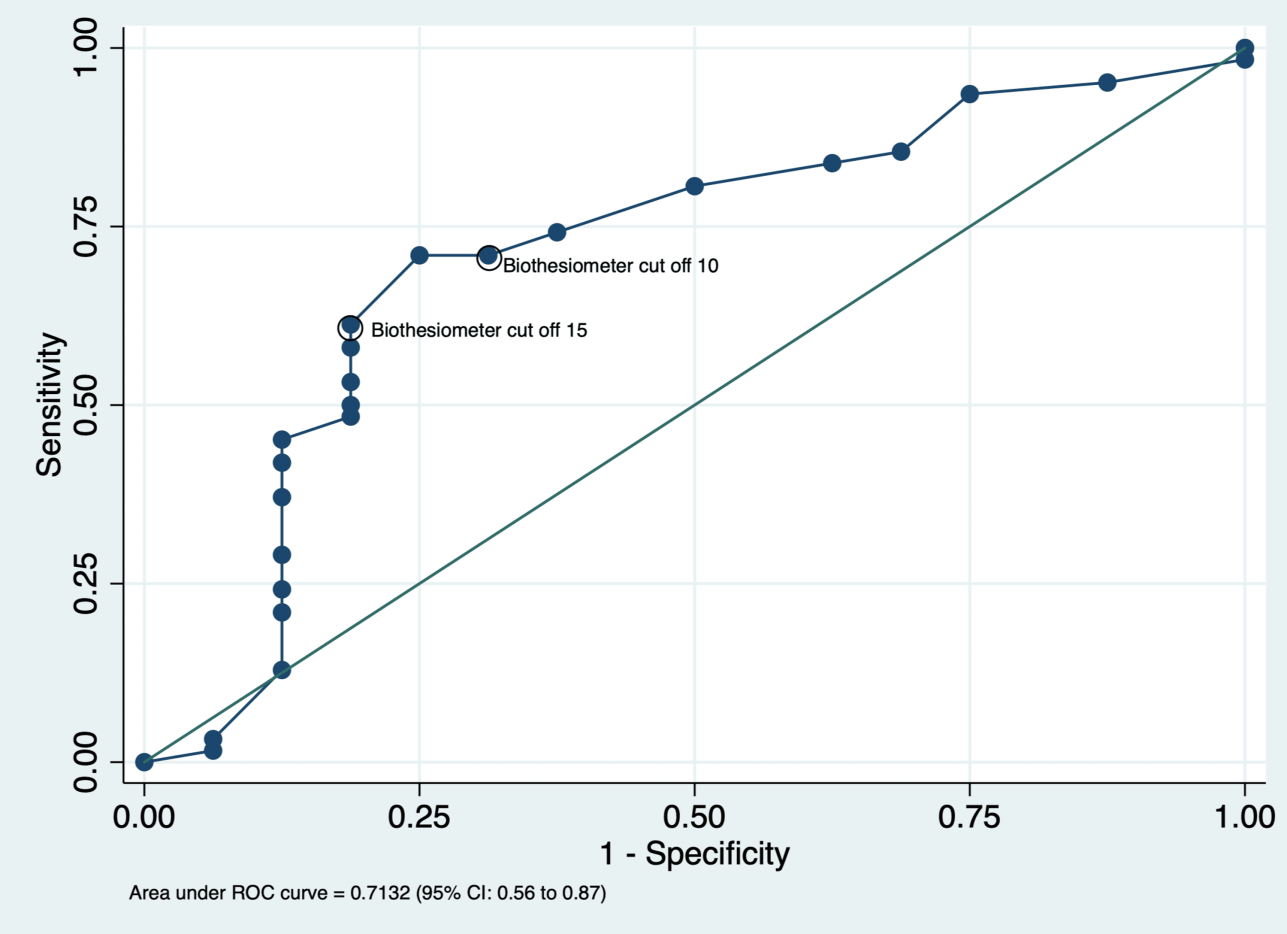
Receiver operating characteristic (ROC) curve for biothesiometry score with reference to nerve conduction studies among patients screened for diabetic peripheral neuropathy.

The ROC curve plotted at each biothesiometry score between true positive and false positive diagnoses showed a score of 10 as the optimum cut-off based on Youden's index. The area under the curve for biothesiometry at that cut-off was 0.73 (0.56 to 0.87) (Figure 2). The sensitivity and specificity of the biothesiometer at various cut-offs are tabulated in the Appendices.

Diagnostic accuracy of various screening methods for screening diabetic neuropathy compared to NCS

Biothesiometry with a cut-off of 10 had the highest sensitivity compared to other methods. The conventional biothesiometry cut-off at 15 had lesser sensitivity compared to the cut-off of 10 (59.4% vs. 71%). However, the predictive values at these two cut-offs did not vary much (positive predictive value: 91.7% vs. 95%). The sensitivity of monofilament and clinical symptoms was 35% and 16%, respectively, if the tests were used as independent screening methods. The composite method using monofilament and biothesiometer >=10 had the highest sensitivity compared to any individual screening method (Tables 3-5).

**Table 3:** Distribution of findings based on the Michigan neuropathy symptom scale compared to nerve conduction studies (n = 39).

Michigan patient symptom score	Nerve conduction studies		
	Abnormal	Normal	Total
Positive	5	0	5
Negative	27	7	34
Total	32	7	39

**Table 4:** Distribution of findings based on the monofilament and biothesiometry compared to nerve conduction studies (N = 78).

Screening tests	Nerve conduction studies		
	Abnormal	Normal	Total
Monofilament			
Positive	22	0	22
Negative	40	16	56
Biothesiometry 10 cut-off			
Positive	44	4	48
Negative	18	12	30
Biothesiometry 15 cut-off			
Positive	31	3	34
Negative	31	13	44

**Table 5:** Diagnostic accuracy of various screening methods for screening diabetic neuropathy compared to nerve conduction studies.

Screening tests	Diagnostic accuracy measure			
	Sensitivity	Specificity	Positive predictive value	Negative predictive value
Michigan patient symptom score, % (95% CI)	15.6% (5.3 to 32.8)	100.0% (59 to 100)	100.0%	20.5% (18.2to 23.1)
Monofilament, % (95% CI)	35.5% (23.7 to 48.7)	100% (79.4 to 100)	100% (84.6 to 100)	28.6% (17.3 to 42.2)
Biothesiometry 10 cut-off, % (95% CI)	71% (58.1 to 81.8)	75% (47.6 to 92.7)	91.7% (80 to 97.7)	40% (22.7 to 59.4)
Biothesiometry 15 cut off, % (95% CI)	50% (37 to 63)	81.3% (54.4 to 96)	91.2% (76.3 to 98.1)	29.5% (16.8 to 45.2)
Composite screening methods (monofilament & biothesiometer 10 cut off)	74.2% (61.5 to 84.5)	75% (47.6 to 92.7)	92% (80.8 to 97.8)	42.9% (24.5 to 62.8)

**Figure 3:**
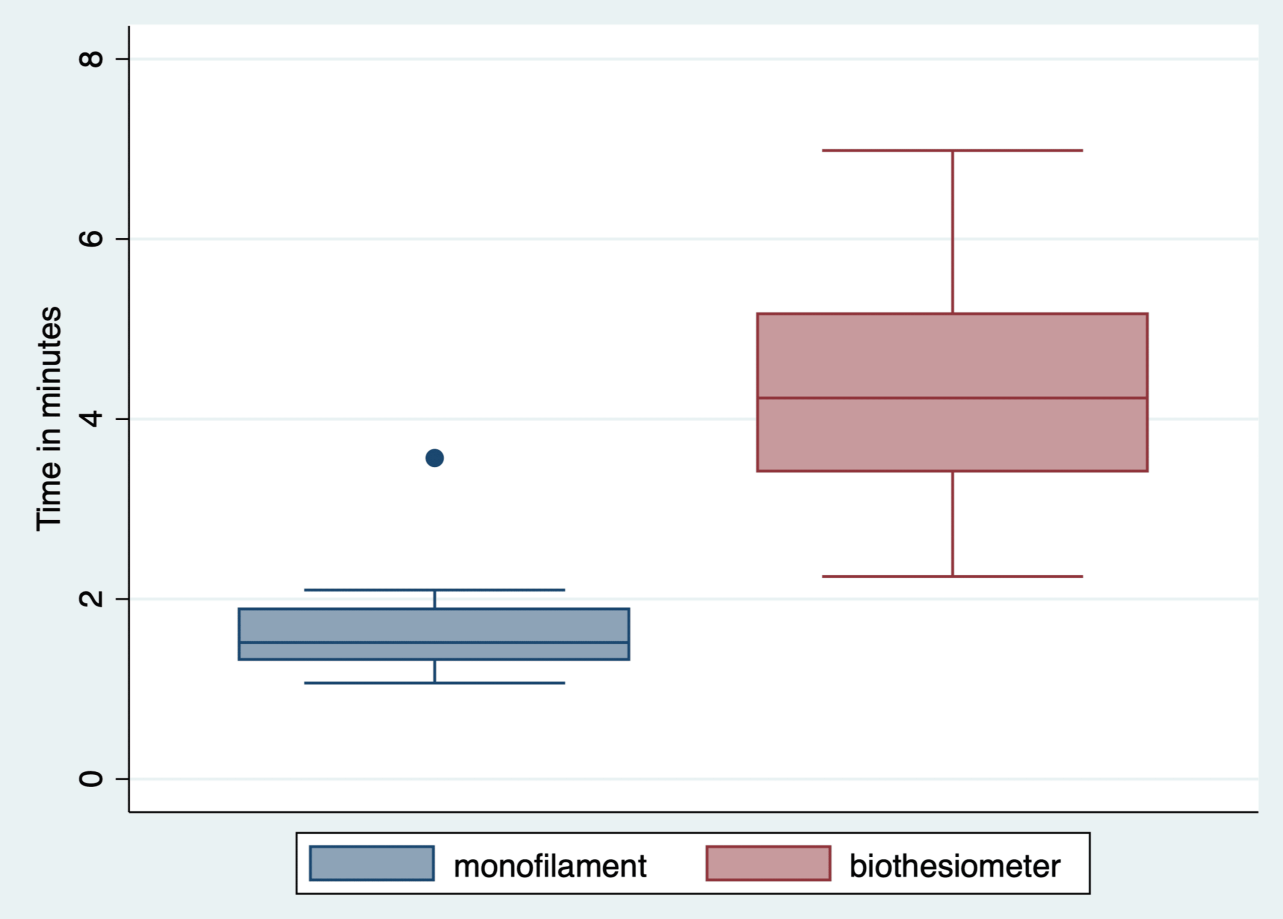
Comparison of time to administer screening methods: monofilament vs. biothesiometer (work sampling). A paired t-test was performed, which yielded a p-value < 0.001.

The mean (SD) time taken for assessment with monofilament was 1.6 (0.5) (95% CI: 1.4 to 1.9) minutes compared to the biothesiometer at 4.4 (1.2) (95% CI: 3.9 to 4.8) minutes. The time taken to administer the biothesiometer was about 2.5 times more compared to the monofilament assessment, which is statistically significant with the p-value of <0.001 (Figure 3). In other words, for every biothesiometer assessment, it requires an additional three minutes compared to the time required for monofilament assessment.

## Discussion

This current study identified that the sensitivity of monofilament and clinical neuropathy score to detect DPN was lower than the biothesiometer. Various systematic reviews on diagnostic accuracy have reported a wide variation in the sensitivity and specificity of the monofilament test. A systematic review and meta-analysis by Wang et al. has reported the sensitivity to vary between 6% and 99% and specificity between 45% and 100% [15]. The pooled sensitivity was estimated to be 53% (95% CI: 32% to 74%) and the pooled specificity was 88% (95% CI: 78% to 94%) [24]. The study by Zhang et al. compared the sensitivity of monofilament based on the number of sites tested, and it did not show much difference in sensitivity or specificity [25].

This variation in sensitivity has been attributed to different sizes of monofilament being used, variations in manufacturers, the number of sites tested, and the location where the test was administered. In addition, after repeated use of monofilament, due to wearing down, the required pressure is not exerted [26]. A recent Indian study found the variability of monofilament based on patient factors such as physical activity, type of footwear, and walking barefoot. Due to these factors, there are wide variations across studies, and they are non-reproducible [27].

In the current study, the monofilament test identified fewer patients with DPN than the biothesiometer. A study from the state of Karnataka by Hazari et al. found an area under the ROC curve of 0.6 for monofilament when biothesiometry was considered as the reference standard [27]. A study by Oyer et al. reported the larger proportions of missing cases with monofilament, though they were positive by VPT [17]. Based on this, they claim the detection of DPN using monofilament will happen at a later stage compared to methods that use vibratory perception. In the study by Jie et al., which compared the diagnostic accuracy of monofilament, VPT, temperature, and tendon reflex, the total positive rate for VPT was 63% as compared to 12% for monofilament, 26.8% for temperature, and 17.2% for tendon reflex [21]. This study used a 10.5 cut-off for biothesiometry based on the cut-off suggested by Hou et al., with NCS as the reference standard [28].

The optimum cut-off for the biothesiometer was identified as 10 in the study population. The sensitivity of the biothesiometer at a cut-off of 15, which is conventionally set by the manufacturers, was lower than the cut-off score identified in the present study [29,30]. An Indian study that assessed the VPT using the biothesiometer among normal healthy volunteers identified 9mv as a cut-off to classify DPN. At this cut-off, 43.3% were identified to have DPN, whereas monofilament identified 6%. This study compares with the current study in terms of the type of monofilament used and operational definitions for classifying DPN [31]. In a study by Mythili et al., where multiple screening methods were compared with NCS, the sensitivity of monofilament was found to be higher than the biothesiometer (98.5% and 86%) [29]. Probably the high cut-off (15mV) used in this study could be the reason for the lower sensitivity of the biothesiometer. A study by Sharma et al. reported the sensitivity of 82% and specificity of 78.8% of the biothesiometer at 15mv cut-off [30].

It is reported that less than 10% of patients with diabetes were screened for DPN [32,33]. The prevalence of DPN can be as high as 80% and more than half of patients with DPN are asymptomatic. To detect the complications earlier, guidelines by the Indian Council of Medical Research emphasized the need for annual foot examinations among diabetics [34]. Widespread awareness is still required for early diagnosis and prevention of foot-related complications of diabetes.

This study has the following strengths. First, to our knowledge, this is one of the very few studies that compared different diagnostic methods with NCS as the gold standard in the Indian context. In this study, we have compared clinical symptom score, monofilament, and biothesiometry with the gold standard test. Second, we did not have any missing data in any of the screening methods. In addition, this study reports additional dimensions related to the time required to administer these different screening tests. Fourth, we kept an independent assessor for monofilament, biothesiometer, and NCS to mask the findings of one screening method from the other teams. Throughout the study, the biothesiometry assessment was performed by one medical technician recruited exclusively for the study purpose. Similarly, the NCS was also performed by a single designated faculty member, who was in charge of the nerve conduction lab. Hence, the scope of having inter-observer variation was avoided. We used the Standards for Reporting of Diagnostic Accuracy Studies (STARD) checklist to report this study.

The study findings should be interpreted with caution, considering the following factors. DPN has a varied spectrum in its natural history, ranging from asymptomatic to DFU. In this study, about half of the patients had complained of at least one symptom in the Michigan neuropathy score. The proportion of people with diabetes diagnosed with DPN is more than 70% in this setting. Since the enrolment happened at a tertiary care facility, which is a referral facility, the majority of the patients could have presented at a late stage of the disease. This may underrepresent cases with early-stage or diabetes without peripheral neuropathy, thereby increasing the chances of selection bias.

As per the hospital policy, NCS is done based on physician requests raised in electronic medical records. For ethical reasons to avoid out-of-pocket expenditure for the patients exclusively for the research purpose, we could not cater to the entire spectrum of patients. For the same reason, we could not do this study with a larger sample size. Tertiary care facilities mostly cater to patients referred from peripheral facilities. Considering the NCS is a paid procedure, it is highly likely that those with severe symptoms and other comorbid conditions would receive the test. That could be the reason for the larger proportions of patients with symptoms in this diagnostic accuracy comparison study. However, it may not be the case in the primary care setting, which may cater to patients with an early spectrum of diabetic neuropathy.

Although we kept an independent assessor for different methods, biothesiometry and Michigan clinical neuropathy scores were conducted by the same assessor. As biothesiometry is an objective score, the knowledge of clinical symptoms is unlikely to influence the biothesiometry score. Further, this study focused on peripheral neuropathy among patients with diabetes. We did not attempt to further classify the etiology of peripheral neuropathy.

Implications

Considering the long-term complications of diabetic foot due to DPN, the current guidelines emphasize the need for screening for diabetic peripheral neuropathy at least once a year or more often based on the risk of foot ulcers among patients with diabetes. However, currently, patients undergoing foot examination are less than 10%.

Despite the low cost of monofilament, the reasons for poor coverage of foot care, such as lack of training and lack of awareness among patients, need to be further explored. Thus, there is a need to emphasize screening for DPN and monitor this activity in routine programs. The VPT score for the biothesiometer varies based on temperature (extreme cold), skin thickness, and the pressure exerted in the probe. Hence, it needs to be confirmed whether the population in the South Asian context needs different cut-offs to detect neuropathy at an early stage.

Moreover, monofilament is felt to be relatively less reliable due to various types of filaments used and a lack of consensus in the number of sites and the type of sites tested. Hence, the recent American Diabetes Association guidelines emphasize the parallel use of at least two screening methods. Considering the additional time required for performing biothesiometry (an additional three minutes for each patient), the feasibility of integrating it into routine outpatient settings needs to be considered. Further, the cost for newer screening methods and NCS is far higher than the simple screening tests, like the monofilament. Though a biothesiometer is reliable, it needs a one-time capital investment, and the trained manpower to administer the test may be a constraint. In resource-constrained settings, the cost-effectiveness of the combined use of monofilament and biothesiometer and justification for testing with monofilament alone should be studied.

## Conclusions

Biothesiometry has higher sensitivity compared to other screening methods, such as monofilament and clinical symptom score. The optimum cut-off level of average cumulative score for biothesiometry against nerve conduction as a gold standard was found to be 10 in people with diabetes. There is a need to emphasize screening for DPN and establish valid cut-offs for biothesiometry to suggest strategies for screening.

## Figures and Tables

**Figure 4:**
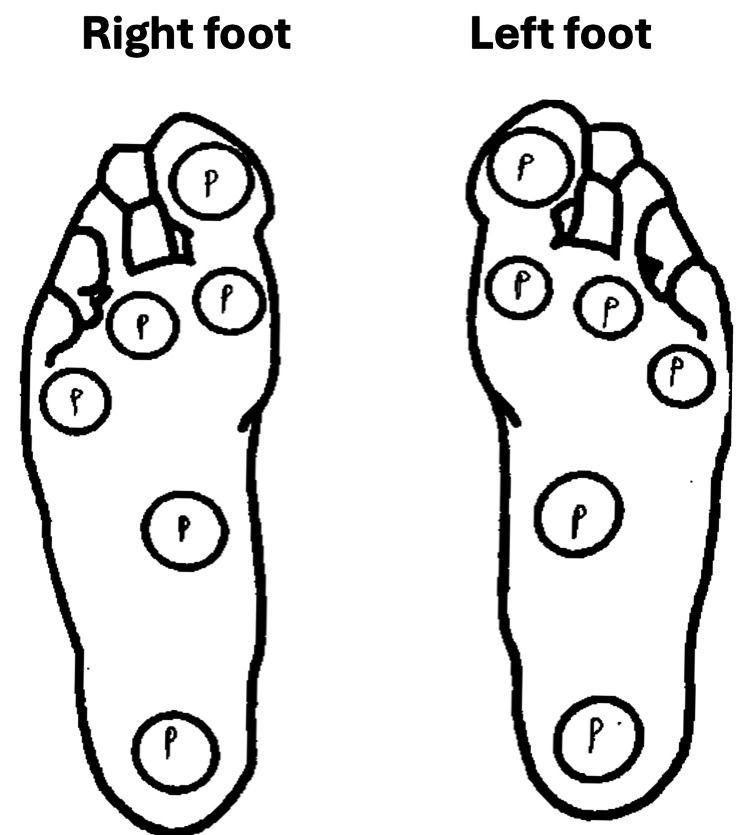
Distribution of sites to be examined in the foot for monofilament test. "P" indicates that there is no peripheral neuropathy. "A" indicates a sign suggestive of peripheral neuropathy.

**Figure 5:**
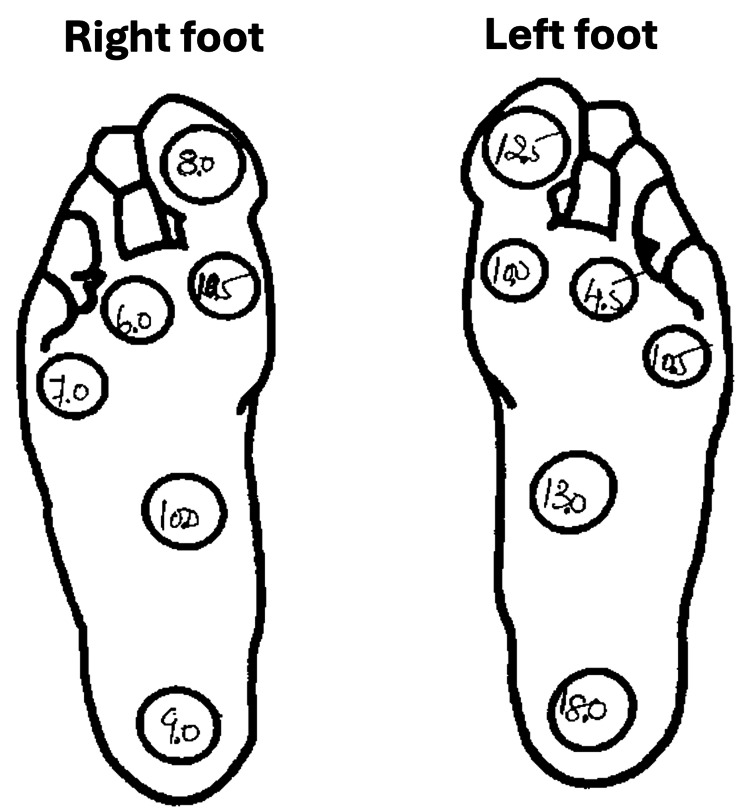
Distribution of sites for the biothesiometry test and scores. Numbers depict scores. Lower scores indicate better nerve conduction.

## Appendices

Performance of the monofilament test

Assessment of Diabetic Neuropathy Based on 10g Monofilament Test (As per the Manufacturer of Monofilament 10g Kodys Guidelines)

Patients were instructed to close their eyes during the assessment of their legs. Before this, they were sensitized to understand the touch sensation of a 10g monofilament by placing it over their hands. Once it was ensured they know how to respond when they feel the touch sensation over various sites in legs, the actual assessment was done at six places over the plantar aspects of legs, namely, the ball of the great toes, the metatarsal head of the first, third, and fifth toes, the middle of the medial border of the foot, and the heel.

Steps in the assessment of neuropathy using monofilament:

1. Ask the patient to lie in the supine position barefoot.

2. Hold the monofilament with thumb and index finger as shown by the manufacturer on the probe.

3. Place the Semmes-Weinstein 10g monofilament (Kodys) perpendicularly over the dorsum of the hand, the middle of the medial border of the foot, and the heel.

4. Ask the patient whether they are able to feel and instruct them to tell yes when they feel a similar kind of sensation in their legs.

5. Ask the patient to close their eyes.

6. Place the monofilament over the plantar aspect of the ball of the great toe.

7. Keep the contact period for at least two seconds till it buckles up to 1 cm.

8. If the patient said yes, mark in the foot diagram as present (P) over the great toe; otherwise, mark it as absent (A).

9. If, for any reason, the great toe was tested, mark it as not sure or not recorded (NR).

10. Similarly, place the monofilament over the other five areas in the plantar aspect of the foot: metatarsal heads of the 1st, 3rd, and 5th toes, the middle of the medial border of the foot, and the heel.

11. Mark these areas according to the instructions mentioned for the great toe.

Classifying the State of At-Risk Foot for Diabetic Peripheral Neuropathy Using the Monofilament Test

1. Assign a score of 1 for each area if the patient is able to feel the sensation of the monofilament.

2. Derive the cumulative score for each leg separately.

3. If the total score is less than or equal to 4 in a foot, it is defined as DPN present, hence the foot is considered as "at risk."

In other words, if the patient is not able to feel the sensation in two or more points in one leg, it was classified as DPN present.

4. Considering the nature of symmetric polyneuropathy in patients with diabetes, if there are signs of DPN in one leg, it is considered as overall DPN present (based on monofilament).

Figure 4 shows the distribution of sites to be examined in the foot for the monofilament test.

Performance of the biothesiometry test

Assessment of Diabetic Neuropathy Based on Biothesiometry

Patients were instructed to close their eyes during the assessment of their legs. Before this, they were sensitized to understand the vibratory sensation by placing the probe over the bony prominence in the hands. Once it was ensured that they knew how to respond when they felt the vibratory sensation over various sites in their legs, the actual assessment was done. It was ensured whether the patient was able to differentiate the vibratory sensation from the pressure sensation. The assessment was done at six places over the plantar aspects of legs, namely, the ball of the great toes, the metatarsal head of the first, third, and fifth toes, the middle of the medial border of the foot, and the heel.

In the leg, the starting vibratory threshold was set with the vibratory sensation threshold perceived over the hands. From there, it was gradually increased till the patient expressed they could feel the vibratory sensation. To confirm the threshold level, again the vibration levels were reduced to see whether the patient is able to feel the vibratory sensation even at the lower threshold level. In each area, the vibratory threshold shown on the screen of biothesiometry was noted.

The average score of vibratory threshold was calculated for each leg (cumulative score/six or number of sites tested). In case any of the site was not assessed for biothesiometry, the average score was estimated based on the number of sites tested. Signs of neuropathy were classified based on the categories specified by the manufacturers (Figure 5).

## References

[1] Deli G, Bosnyak E, Pusch G, Komoly S, Feher G (2013). Diabetic neuropathies: diagnosis and management. Neuroendocrinology.

[2] Perveen W, Ahsan H, Rameen Shahzad (2024). Prevalence of peripheral neuropathy, amputation, and quality of life in patients with diabetes mellitus. Sci Rep.

[3] Sun J, Wang Y, Zhang X, Zhu S, He H (2020). Prevalence of peripheral neuropathy in patients with diabetes: a systematic review and meta-analysis. Prim Care Diabetes.

[4] Rodrigues BT, Vangaveti VN, Urkude R, Biros E, Malabu UH (2022). Prevalence and risk factors of lower limb amputations in patients with diabetic foot ulcers: a systematic review and meta-analysis. Diabetes Metab Syndr.

[5] Zhang P, Lu J, Jing Y, Tang S, Zhu D, Bi Y (2017). Global epidemiology of diabetic foot ulceration: a systematic review and meta-analysis. Ann Med.

[6] Jeffcoate WJ, Harding KG (2003). Diabetic foot ulcers. Lancet.

[7] Chen L, Sun S, Gao Y, Ran X (2023). Global mortality of diabetic foot ulcer: a systematic review and meta-analysis of observational studies. Diabetes Obes Metab.

[8] Saluja S, Anderson SG, Hambleton I (2020). Foot ulceration and its association with mortality in diabetes mellitus: a meta-analysis. Diabet Med.

[9] ElSayed NA, Aleppo G, Aroda VR (2023). Retinopathy, neuropathy, and foot care: standards of care in diabetes—2023. Diabetes Care.

[10] Selvarajah D, Kar D, Khunti K, Davies MJ, Scott AR, Walker J, Tesfaye S (2019). Diabetic peripheral neuropathy: advances in diagnosis and strategies for screening and early intervention. Lancet Diabetes Endocrinol.

[11] Atmaca A, Ketenci A, Sahin I (2024). Expert opinion on screening, diagnosis and management of diabetic peripheral neuropathy: a multidisciplinary approach. Front Endocrinol (Lausanne).

[12] Carmichael J, Fadavi H, Ishibashi F, Shore AC, Tavakoli M (2021). Advances in screening, early diagnosis and accurate staging of diabetic neuropathy. Front Endocrinol (Lausanne).

[13] Herman WH, Pop-Busui R, Braffett BH, Martin CL, Cleary PA, Albers JW, Feldman EL (2012). Use of the Michigan Neuropathy Screening Instrument as a measure of distal symmetrical peripheral neuropathy in type 1 diabetes: results from the Diabetes Control and Complications Trial/Epidemiology of Diabetes Interventions and Complications. Diabet Med.

[14] Petropoulos IN, Ponirakis G, Khan A, Almuhannadi H, Gad H, Malik RA (2018). Diagnosing diabetic neuropathy: something old, something new. Diabetes Metab J.

[15] Wang F, Zhang J, Yu J (2017). Diagnostic accuracy of monofilament tests for detecting diabetic peripheral neuropathy: a systematic review and meta-analysis. J Diabetes Res.

[16] Dros J, Wewerinke A, Bindels PJ, van Weert HC (2009). Accuracy of monofilament testing to diagnose peripheral neuropathy: a systematic review. Ann Fam Med.

[17] Oyer DS, Saxon D, Shah A (2007). Quantitative assessment of diabetic peripheral neuropathy with use of the clanging tuning fork test. Endocr Pract.

[18] Duke J, McEvoy M, Sibbritt D, Guest M, Smith W, Attia J (2007). Vibrotactile threshold measurement for detecting peripheral neuropathy: defining variability and a normal range for clinical and research use. Diabetologia.

[19] Mooi CS, Lee KW, Yusof Khan AH (2024). Using biothesiometer, Neuropathy Symptom Score, and Neuropathy Disability Score for the early detection of peripheral neuropathy: a cross-sectional study. Qatar Med J.

[20] Ghosal S, Stephens J, Mukherjee A (2012). Quantitative vibration perception threshold in assessing diabetic neuropathy: is the cut-off value lower for Indian subjects? [Q-VADIS Study]. Diabetes Metab Syndr.

[21] Jie FY, Zafar MI, Xu L, Shafqat RA, Gao F (2018). Sensitivity of four simple methods to screen Chinese patients for diabetic peripheral neuropathy. Acta Endocrinol (Buchar).

[22] Feldman EL, Stevens MJ, Thomas PK, Brown MB, Canal N, Greene DA (1994). A practical two-step quantitative clinical and electrophysiological assessment for the diagnosis and staging of diabetic neuropathy. Diabetes Care.

[23] Giacomini PS (2006). Electromyography and neuromuscular disorders: clinical electrophysiologic correlations. Mcgill J Med.

[24] Feng Y, Schlösser FJ, Sumpio BE (2009). The Semmes Weinstein monofilament examination as a screening tool for diabetic peripheral neuropathy. J Vasc Surg.

[25] Zhang Q, Yi N, Liu S (2018). Easier operation and similar power of 10 g monofilament test for screening diabetic peripheral neuropathy. J Int Med Res.

[26] McGill M, Molyneaux L, Spencer R, Heng LF, Yue DK (1999). Possible sources of discrepancies in the use of the Semmes-Weinstein monofilament. Impact on prevalence of insensate foot and workload requirements. Diabetes Care.

[27] Hazari A, Mishra V, Kumar P, Maiya A (2024). The accuracy of 10 g monofilament use for clinical screening of diabetes peripheral neuropathy among Indian population. PLoS One.

[28] Hou Y, Liu S, Zhu T, Zhang H, Liu G, Zhu Y, Chen H (2012). Vibration perception threshold in diagnosing diabetic peripheral neuropathy by receiver operating characteristic curve. (Article in Chinese). Zhong Nan Da Xue Xue Bao Yi Xue Ban.

[29] Mythili A, Kumar KD, Subrahmanyam KA, Venkateswarlu K, Butchi RG (2010). A comparative study of examination scores and quantitative sensory testing in diagnosis of diabetic polyneuropathy. Int J Diabetes Dev Ctries.

[30] Sharma KNS, Kumar HA (2023). Assessment of the diagnostic accuracy of Vibrasense compared to a biothesiometer and nerve conduction study for screening diabetic peripheral neuropathy. J Foot Ankle Res.

[31] Gill HK, Yadav SB, Ramesh V, Bhatia E (2014). A prospective study of prevalence and association of peripheral neuropathy in Indian patients with newly diagnosed type 2 diabetes mellitus. J Postgrad Med.

[32] Selvaraj K, Ramaswamy G, Radhakrishnan S, Thekkur P, Chinnakali P, Roy G (2016). Self-care practices among diabetes patients registered in a chronic disease clinic in Puducherry, South India. J Soc Health Diabetes.

[33] Saurabh S, Sarkar S, Selvaraj K, Kar SS, Kumar SG, Roy G (2014). Effectiveness of foot care education among people with type 2 diabetes in rural Puducherry, India. Indian J Endocrinol Metab.

[34] Indian Council of Medical Research (2018). ICMR Guidelines for Management of Type 2 Diabetes 2018. https://www.icmr.gov.in/icmrobject/custom_data/pdf/resource-guidelines/ICMR_GuidelinesType2diabetes2018_0.pdf.

